# Regulation of Toll-Like Receptors-Mediated Inflammation by Immunobiotics in Bovine Intestinal Epitheliocytes: Role of Signaling Pathways and Negative Regulators

**DOI:** 10.3389/fimmu.2014.00421

**Published:** 2014-09-02

**Authors:** Julio Villena, Hisashi Aso, Haruki Kitazawa

**Affiliations:** ^1^Immunobiotics Research Group, Tucuman, Argentina; ^2^Laboratory of Immunobiotechnology, Reference Centre for Lactobacilli (CERELA-CONICET), Tucuman, Argentina; ^3^Cell Biology Laboratory, Graduate School of Agricultural Science, Tohoku University, Sendai, Japan; ^4^Food and Feed Immunology Group, Laboratory of Animal Products Chemistry, Graduate School of Agricultural Science, Tohoku University, Sendai, Japan

**Keywords:** immunobiotics, TLR4, intestinal immunity, inflammation, bovine intestinal epitheliocytes, TLR negative regulators, lactobacilli, bifidobacteria

## Abstract

Intestinal epithelial cells (IECs) detect bacterial and viral associated molecular patterns via germline-encoded pattern-recognition receptors (PRRs) and are responsible for maintaining immune tolerance to the communities of resident commensal bacteria while being also capable to mount immune responses against pathogens. Toll-like receptors (TLRs) are a major class of PRRs expressed on IECs and immune cells, which are involved in the induction of both tolerance and inflammation. In the last decade, experimental and clinical evidence was generated to support the application of probiotics with immunoregulatory capacities (immunobiotics) for the prevention and treatment of several gastrointestinal inflammatory disorders in which TLRs exert a significant role. The majority of these studies were performed in mouse and human cell lines, and despite the growing interest in the bovine immune system due to the economic importance of cattle as livestock, only few studies have been conducted on cattle. In this regard, our group has established a bovine intestinal epithelial (BIE) cell line originally derived from fetal bovine intestinal epitheliocytes and used this cell line to evaluate the impact of immunobiotics in TLR-mediated inflammation. This review aims to summarize the current knowledge of the beneficial effects of immunobiotics in the regulation of intestinal inflammation/infection in cattle. Especially, we discuss the role of TLRs and their negative regulators in both the inflammatory response and the beneficial effects of immunobiotics in bovine IECs. This review article emphasizes the cellular and molecular interactions of immunobiotics with BIE cells through TLRs and gives the scientific basis for the development of immunomodulatory feed for bovine healthy development.

## Introduction

Intestinal epithelial cells (IECs) are structurally and functionally polarized. These cells have an apical surface facing the intestinal lumen and a basolateral surface facing the underlying basement membrane and the lamina propria. IECs provide a physical barrier that separates commensal bacteria in the lumen from the underlying lamina propria and deeper intestinal layers (1). In addition, IECs are a central component of the immune system of the gut. Over the last decades, great progress has been achieved in understanding IECs immunobiology (2). It was amply demonstrated that the cross-talk between the epithelium with gut microbes significantly influences the activities of immune cells in the mucosa (2). The detection of commensal bacteria, pathogens, or probiotics by IECs is achieved through the families of germline-encoded pattern-recognition receptors (PRRs) that recognize conserved molecular structures known as microbe-associated molecular patterns (MAMPs). MAMPs and PRRs interaction and the subsequent signaling in IECs is involved in several important mechanisms that are crucial for maintaining a healthy epithelial barrier including maintenance of tight junctions strength, epithelial cell proliferation and renewal, expression of antimicrobial peptides, and modulation of mucosal immune responses (3).

In recent years, worldwide interest has rapidly and significantly increased in the therapeutic and preventive effects of “friendly bacteria.” These microorganisms, recognized as probiotics, are generally selected from *Lactobacilli* or *Bifidobacteria* strains (4). Several studies in animal models as well as clinical trials support a unique role for probiotics by beneficially modulating the mucosal immune system. Thus, a new term was required to identify probiotic bacteria that promote health by regulating the mucosal immune system. Clancy suggested the new term “immunobiotics” as appropriate for fulfilling this need (5). The quest for a better understanding of how immunobiotics works have led to an enormous interest in the molecular processes underlying host–microbe interactions. As reviewed by Lebeer et al. (6), the final conclusion of works that have studied the molecular mechanism of probiotic immunomodulatory activities is that: “*their effect depends on the combination of distinct MAMPs that interact with various PRRs and the associated co-receptors that fine tune signaling, as well as on the quantity and quality of these MAMPs. Therefore, host-immunobiotic interactions are not univocal but involve complex interactions among various microbial molecules, host receptors, and adaptor molecules*” (6).

This expanding knowledge about the cellular and molecular effects of beneficial bacteria in innate mucosal immune system has raised the possibility of new treatments for improving health not only in humans but also in animals. In this review, we describe the recent advances in the impact of immunobiotics on bovine intestinal epithelial (BIE) cells and possible novel therapeutic approaches to beneficially modulate bovine epithelial cell immunobiology. Especially, we discuss the role of toll-like receptors (TLRs), their signaling pathways, and their negative regulators in both the inflammatory-intestinal injury and the beneficial effects of immunobiotics. This article emphasizes the cellular and molecular interactions of immunobiotics with bovine epithelial cells through TLRs and gives the scientific basis for the future development of immunomodulatory feed for improving bovine health.

## Bovine Intestinal Epithelial Cells

The development of bovine intestinal cell cultures and their characterization with regard to their permissiveness for bacterial adhesion and invasion, and the ability to sense PAMPs through PRRs represents an important step forward toward the establishment of *in vitro* systems to study molecular interactions of pathogenic, commensal, and probiotic microorganisms with the bovine host (7).

For cattle, primary cultures of ileum or colon epithelial cells have been used for toxicological assays, the study of microbial virulence factors, the efficacy of antimicrobial compounds, and the evaluation of innate immune responses through PRRs signaling (7–12). A combination of the enzymatic digestion (dispase and collagenase) together with soft mechanical agitation proved to be a successful method for releasing intact, viable bovine colonic crypts from underlying mesenchymal tissue. However, a series of purification steps was required to eliminate the majority of contaminating non-epithelial cells (mostly fibroblasts) from the crypt suspension (10). Similarly, cultures of bovine colonocytes and jejunocytes were obtained by Rusu et al. (11), using a combination of enzymatic and mechanical disruption of the intestinal epithelium. The study showed that primary cultures of bovine enterocytes isolated from colon and jejunum presented characteristics of epithelial cells, such as a typical pavement-like aspect, the formation of domes and apical tight junctions, and microvilli in confluent cultures. Moreover, these bovine colonocytes and jejunocytes expressed epithelial cell markers such as brush border enzymes and the epithelium typical cytoskeleton proteins, such as cytokeratins.

Dibb-Fuller et al. (8) developed primary bovine cell lines from ileum, colon, and rectum and those bovine primary gastrointestinal epithelial-derived cells were successfully used to assess adherence and invasion of several intestinal pathogenic bacteria including enterohemorrhagic *Escherichia coli* (EHEC) and *Salmonella enterica* serotype typhimurium. In addition, bovine colonic crypts cells isolated and purified from the mucosa, proved to be useful *in vitro* tools to study virulence factors of EHEC, verotoxins, in particular (10). The work showed the expression of globotriaosylceramide Gb3 by bovine colonocytes, which directly contrasts with the absence of this receptor on human intestinal epithelium. This fact represents a fundamental difference that could have major significance in the different pathogenicity of EHEC in these hosts, and make bovine colonocytes an invaluable tool for studying EHEC infection (10).

Bridger et al. (7) showed that epithelioid cells from bovine colonic crypts formed a confluent monolayer on the surface of collagen-coated culture flasks. Those bovine colonocytes expressed epithelial cell-specific cytokeratin and cell membrane-associated tight junctional ZO-1 in the contact area between neighboring cells. Semi-quantitative RT-PCR demonstrated variable amounts of gene transcripts for different TLR genes. Notably, primary bovine colonocytes expressed TLR4 mRNA while transcripts for TLR1, TLR3, and TLR6 were also detectable in some cultures. Moreover, the study showed that colonocytes significantly up-regulated the expression of IL-8, MCP-1, and RANTES when challenged with pathogenic *E. coli* or lipopolysaccharide (LPS) (7). Therefore, short-term bovine colonocytes cultures proved to be suitable *in vitro* models to study pathogens-specific responses of the bovine colonic mucosa.

Those studies clearly demonstrated that bovine primary IEC cultures represent valuable tools to assess the molecular mechanisms involved in pathologies caused by infectious agents. However, the cellular and molecular interactions of commensal or probiotic bacteria with BIE cells have been less explored.

There is an increasing research in the use of immunobiotics to beneficially modulate the mucosal immune system in animals. Immunobiotic bacteria could be used for improving resistance against pathogens and decreasing intestinal inflammatory-mediated tissue damage (13–17). In this regard, our laboratories and others’ have conducted *in vitro* and *in vivo* studies utilizing different lactobacilli and bifidobacteria strains to evaluate the effect of immunobiotics against infections and inflammation in animals, however, the majority of these studies were performed in swine and only few in the cattle (13).

We hypothesized that a cell line of BIE cells could be a useful *in vitro* model for the evaluation of the molecular interactions between IECs and bovine pathogens. In addition, such system would allow the selection of immunobiotic microorganisms and the study of the mechanisms by which probiotic lactic acid bacteria (LAB) functionally modulate bovine IECs. Therefore, in order to: (a) understand the functional role of IECs in bovine mucosal host defense and (b) select potential immunobiotic LAB strains that may be used to beneficially modulate the inflammatory response in bovine IECs; we have recently established an immortalized BIE cell line (18). Monolayer cobblestone and epithelial-like morphology is assumed by BIE cells when cultured, with close contact between the cells. Moreover, scanning electron microscopy examination of BIE cell reveled that 3-days old cells have irregular and slender microvilli-like structures on their surface and that this structures increase in complexity as the cells grow (18). BIE cells undergoes over at least 40 passages with no detectable loss of epithelial properties.

**Figure 1:**
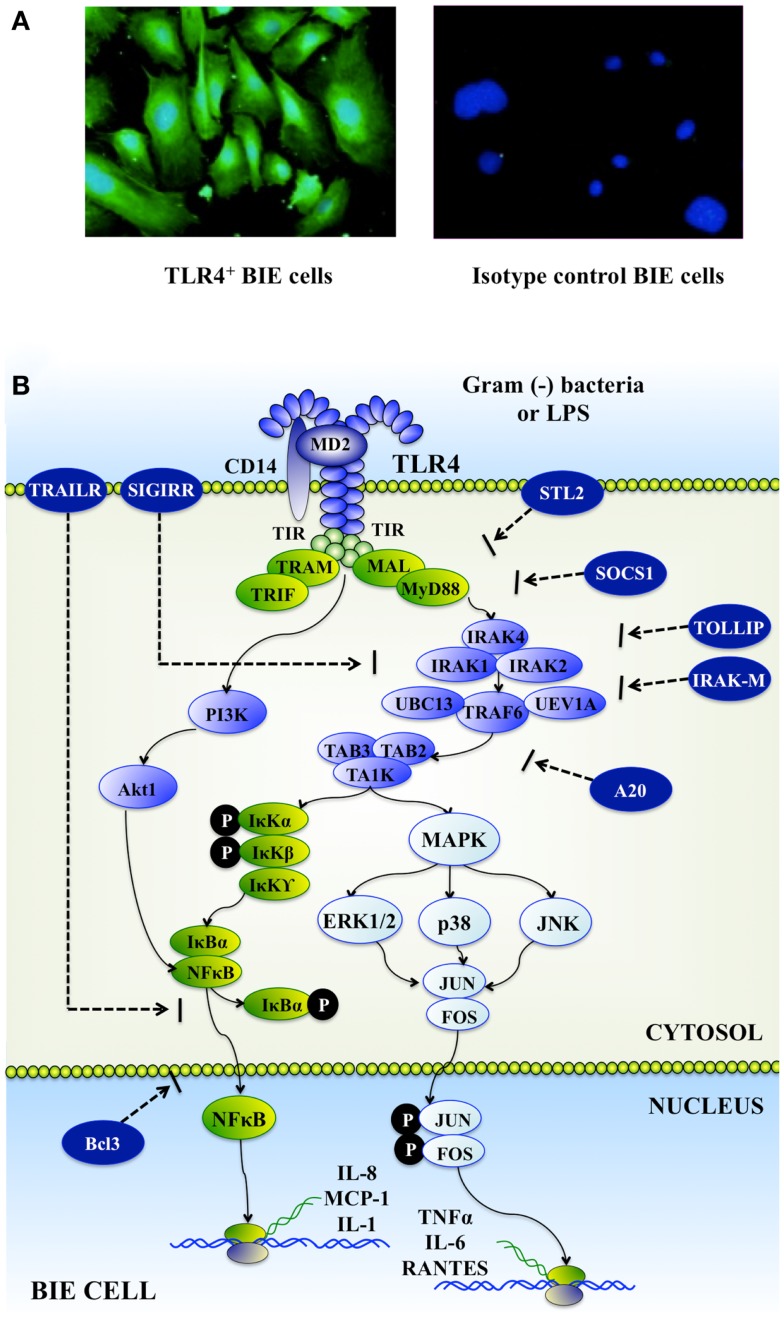
**Toll-like receptor 4 (TLR4) expression in bovine intestinal epithelial epitheliocytes (BIE cells) (A)**. Upon recognition of lipopolysaccharide (LPS), TLR4 dimerizes and initiates a signaling cascades that include phosphatidylinositol 3-kinase (PI3K), mitogen-activated protein kinase (MAPK), and nuclear factor κB (NF-κB) pathways; and culminates in the production of inflammatory mediators by BIE cells **(B)**.

Expression of TLRs mRNA in BIE cells was evaluated by RT-qPCR and it was demonstrated that all TLRs genes were expressed in BIE cells (19). TLR1, 3, 4, and 6 were strongly expressed, followed by TLR5, 8, 9, 10, 2, and 7. We were particularly interested in expression of TLR2 and TLR4 in BIE cells as the main receptors detecting Gram(+) probiotic bacteria and Gram(−) pathogens, respectively. Therefore, to confirm real-time PCR findings, we further examined the expression of TLR2 and 4 proteins in BIE cells using anti-TLRs antibodies that are able to cross-react with bovine TLRs (19). Visualization of the immunofluorescence staining confirmed the protein expression of TLR2 and 4 in BIE cells (Figure 1A).

The inflammatory response triggered by BIE cells in the face of a challenge with heat-stable enterotoxigenic *E. coli* (ETEC) MAMPs was also evaluated. Upon pathogen binding to TLR4 complex, this receptor recruits, through its short intracellular toll-interleukin-1 receptor (TIR) domain, adaptor molecules, and kinases, thus initiating a downstream signaling cascade that culminates in the production and secretion of inflammatory mediators such as TNF-α, IL-1β, IL-6, and IL-8 (Figure 1B). The ETEC 987P strain used in our experiments does not express the TLR5-ligand flagellin, and we demonstrated that the main molecule responsible for the inflammatory response triggered by this bacterium is the LPS (15, 16). Stimulation of BIE cells with heat-stable ETEC MAMPs from strain 987P enhanced the production of the pro-inflammatory cytokines IL-6, IL-8, IL-1β, and MCP-1 by activating mitogen-activated protein kinase (MAPK) and nuclear factor κB (NF-κB) pathways (19). These findings are in line with our previous reports demonstrating that the heat-killed ETEC 987P strain triggers a TLR4-mediated inflammatory response in porcine intestinal epithelial (PIE) cells through NF-κB and MAPK pathways (20). In addition, our results in BIE cells correlate with studies of the immune response against ETEC in IECs of different hosts’ species. It was shown that both NF-κB and MAPK pathways are important mediators of ETEC and LPS activation in human (HT-29 and T84) and mouse (CMT93) IECs (15, 21).

Available lines of evidence indicate that bovine epithelial cells, including intestinal, mammary, bronchial, and nasopharynx epithelial cells respond to bacterial LPS and other microbial products by producing pro-inflammatory cytokines required to combat invading pathogens. Therefore, the pro-inflammatory mediators produced by BIE cells in response to ETEC may have an important protective role during the course of intestinal infections. The chemokine IL-8 stimulates a strong infiltration of neutrophils in the gut lamina propria, a fact that is consistently observed upon ETEC infection. After IL-8 induced recruitment of neutrophils, increase of IL-6 production is able to induce degranulation of these cells, thereby enhancing the inflammatory response (22). In addition, IECs are able to produce MCP-1 in response to ETEC challenge. This chemokine has potent monocyte-activating and attracting properties and plays a major role during intestinal inflammation (23). Therefore, BIE cells respond to the presences of ETEC and LPS by activating the TLR4-signaling pathway, which is necessary to initiate a robust defensive action against intruders.

Our studies indicate then that the BIE cell line could be a useful cell line for evaluating inflammatory responses via TLR4 *in vitro*. Furthermore, considering that inflammatory responses induced by intestinal pathogens can lead to dysregulation of IECs signaling, disruption of membrane barrier integrity, enhancement of pathogen translocation and disease (24), BIE cells could be also used to evaluate therapies designed for preventing inflammatory damage caused by bovine intestinal pathogens or their associated PAMPs or virulence factors.

## Probiotics for the Bovine Host

Several studies on the pathogenesis of intestinal inflammation/infection both in man and experimental animals continue to show the importance of commensal bacteria in the gastrointestinal tract in stimulating and directing the immune system. Moreover, the ability of immunobiotic bacteria to beneficially modulate the response against intestinal pathogens in animals through the improvement of resistance and the reduction of inflammatory-mediated tissue damage has been described by several reports (25–27). Before weaning, dairy calves are highly susceptible to several pathogens. For several years, antibiotics have been used to overcome these problems also to obtain economic benefits in terms of improved calves performance and reduced medication costs. However, the use of antibiotics in animal husbandry is in question because of antibiotic resistance of microorganisms. In an effort to replace antibiotics from bovine feeds, many additives have been proposed including the use of probiotics (28, 29). In fact, some few studies have shown that probiotic bacteria can be used as growth promoters in calves instead of antibiotics to counteract the negative effects of their widespread use (30). Early studies of Abe et al. (31) showed that oral administration of *Bifidobacterium pseudolongum* or *Lactobacillus acidophilus* to calves improved body weigh gain and decreased the frequency of diarrhea occurrence. Similarly, Mokhber-Dezfouli et al. (32) demonstrated that probiotic treatments have the ability to beneficially modulate body weight gain, body height, and general health condition of calves. Additionally, oral treatment with probiotic *E. coli* significantly reduced the pathogenicity and fecal shedding of EHEC in calves (33, 34). It was also reported that a mixture composed of *Lactobacillus casei* DSPV 318T, *Lactobacillus salivarius* DSPV 315T, and *Pediococcus acidilactici* DSPV 006T protected calves against *Salmonella* Dublin infection (35). These studies clearly show the potential of probiotic bacteria to beneficially modulate gastrointestinal hemostasis in the bovine host. However, the cellular and molecular mechanisms involved in the probiotic activities in cattle have not been studied in depth.

## Regulation of Inflammation in BIE Cells by Immunobiotic Lactobacilli

The first contact of immunobiotic bacteria with the intestinal mucosa is mediated by the single cell layer of IECs. As mentioned before, these IECs are of paramount importance in host–immunobiotic cross-talk. Then, we thus sought to determine whether an immunobiotic *Lactobacillus* strain could regulate the inflammatory response induced by heat-stable ETEC MAMPs in BIE cells. Our previous studies with the strain *Lactobacillus jensenii* TL2937 showed that this bacterium has remarkable immunomodulatory effects in porcine IECs and immune cells [for a review see Ref. (3)]. The TL2937 strain is able to functionally modulate porcine IECs by inhibiting excessive MAPK- and NF-κB-induced pro-inflammatory cytokine production (IL-6 and IL-8) in response to TLR4 activation (3, 15). Consequently, we first focused on *L. jensenii* TL2937 to evaluate its anti-inflammatory effect in BIE cells. Preincubation of BIE cells with *L. jensenii* TL2937 significantly decreased IL-6 and IL-8 expressions in 20 and 25% with respect to the control, respectively, after heat-stable ETEC MAMPs challenge (19). However, this effect was lower when compared with the anti-inflammatory activity of this strain in PIE cells (15). In porcine, IECs previously treated with the TL2937 strain, stimulation with heat-stable ETEC MAMPs reduced IL-6 and IL-8 expressions by 35 and 30% when compared to control cells, respectively (15). Although the effect of the *L. jensenii* TL2937 in BIE cells was lower than the one previously reported for porcine IECs, our first studies in BIE cells indicated that probiotic lactobacilli could be beneficial for attenuating inflammatory damage caused by TLR4 activation in bovine epithelial cells (19). Thus, we next aimed to screen and select the most effective immunoregulatory lactobacilli strains able to modulate TLR4-mediated pro-inflammatory response in BIE cells. Several lactobacilli strains were evaluated in our bovine IECs line and we found that some of these bacteria were capable to downregulate the expression of inflammatory cytokines. Among these strains, *L. casei* OLL2768 showed the most pronounced effect (19). Notably, the anti-inflammatory activity of the OLL2768 strain was more pronounced than that observed for *L. jensenii* TL2937 in BIE cells, while the effect of OLL2768 strain was lower in PIE cells (15). It is well known that probiotic activities are strain specific. In addition, our findings clearly indicated that is necessary to carefully evaluate different strains according to the specific host, because the effect of the same *Lactobacillus* may be different according to the host that consumes it. Then, our *in vitro* bovine system could be of great value to find potential immunobiotic strains suitable for the improvement of the bovine host health.

We also aimed to define the molecular mechanisms by which *L. casei* OLL2768 attenuated heat-stable ETEC MAMPs-induced pro-inflammatory response in BIE cells. Our data showed that the immunoregulatory effect was related to the capacity of OLL2768 strain to inhibit NF-κB and MAPK p38 signaling pathways in bovine IECs after TLR4 activation.

Nuclear factor κB is composed of several protein subunits regulating DNA transcription. Under non-stimulatory conditions, it is bound to the inhibitor molecule IkB in the cytoplasm. After TLR activation IkB is phosphorylated by IKK and once freed from IkB, NF-κB subunit p65 (RelA) migrates into the nucleus, where it binds to target promoters and activates transcription of effector genes including TNF-α, IL-8, and others (36, 37). Among many up-stream signaling proteins involved in NF-κB activation, TLR4 plays a critical role and it is well-documented that TLR4/NF-κB pathway has a pivotal role in the pathogenesis of several intestinal inflammatory diseases and infection-induced tissue damage (38). Some studies have reported the ability of probiotic lactobacilli to modulate TLR4/NF-κB pathway in the gut (39, 40). It was showed that *Lactobacillus suntoryeus*, a gut commensal, blocks inflammatory mediators (Cox2, TNF-α, IL-1, and IL-6) through suppression of TLR4-linked NF-κB activation in mice with 2,4,6-trinitrobenzene sulfonic acid-induced colitis (41). Liu et al. (42) reported that *Lactobacillus reuteri* strains DSM 17938 and ATCCPTA4659 led to decrease intestinal protein levels of TLR4 and decreased pro-inflammatory cytokine levels in parallel with inhibition of TLR4-signaling via the NF-κB pathway in newborn rats with necrotizing enterocolitis. In addition, it was reported that some probiotics strains are able to suppress TNF- or *Salmonella typhimurium*-induced IL-8 gene expression and secretion by IECs in a NF-κB-dependent manner (39, 40). Our experiments also demonstrated that *L. casei* OLL2768 is able to inhibit TLR4/p38 signaling pathway since we demonstrated that in lactobacilli-treated BIE cells the phosphorylation of p38 was reduced after challenge in heat-stable ETEC MAMPs (19). Regulation of MAPK p38 pathway by probiotics has been described before. *L. rhamnosus* GG was found to significantly down-regulate expression of p38 in human monocyte-derived DCs after the challenge with LPS (43). It was also showed that *Lactobacillus bulgaricus* LBG, inhibited the activation of the TLR4-signaling pathway and IL-8 production induced by *Helicobacter pylori* LPS in human gastric adenocarcinoma cells through blocking MAPK p38 (44). Then, our findings in BIE cells are reminiscent of other studies showing that probiotic *L. casei* OLL2768 is capable of modulating TLR4/NF-κB- and TLR4/p38-induced inflammation.

The JNK and p38 MAPK pathways share several up-stream regulators, and accordingly there are multiple stimuli that simultaneously activate both pathways. In this regard, it was showed that the conditioned media from probiotic *L. rhamnosus* GG induced the expression of cellular heat shock protein (Hsp72) in IECs in a p38- and JNK-dependent manner (45). The work showed that *L. rhamnosus* GG conditioned media treatment resulted in a clear activation of both p38 and JNK pathways in IECs. Moreover, exposure of IECs to inhibitors against p38 and JNK before conditioned media treatment resulted in blockade of Hsp72 expression, thus, confirming a likely role for both MAPK signaling pathways in the probiotic effect (45). Then, we expected that *L. casei* OLL2768 had an inhibitory effect on JNK pathway in BIE cells as observed for the p38 MAPK pathway. However, increased levels of p-JNK were detected in BIE cells stimulated with the OLL2768 strain. It was also reported that JNK and p38 MAPK pathways may induce opposite effects. In fact, there is evidence indicating that the p38 MAPK pathway can negatively regulate JNK activity in several contexts (46, 47). The first evidence of the interaction of these two pathways was the observation that inhibition of p38 strongly increased the activation of JNK (46). The work analyzed the effect of specific p38 MAPK inhibitors, SB202190 or SB203580, on JNK phosphorylation in A549 human lung alveolar epithelial cells, and found that inhibition of p38 MAPK could induce JNK activation due to a compensatory mechanism (46). In addition, it was showed that the p38 inhibitor SB203580 enhances the activation of JNK isoforms after the challenge of IECs with IL-1β or by LPS in macrophages (46). In line with those studies, the kinetic analysis of p38 and JNK phosphorylation in BIE cells showed an early up-regulation of p–p38 between 5 and 10 min after heat-stable ETEC MAMPs challenge that was followed by a down-regulation of p-JNK between 10 and 20 min (19). Therefore, we can speculate that *L. casei* OLL2768 has a direct influence in p38 pathway while its effect in JNK is the result of the inhibition of p38 phosphorylation. Further research is needed to clarify completely the influence of *L. casei* OLL2768 in MAPK pathways in BIE cells.

Following TLR activation, there must be a checkpoint where TLR signaling is abolished and the system is returned to a normal physiological state to avoid a harmful response toward the host immune system. Regulatory factors able to modulate the duration and intensity of TLRs signals are therefore key components for the protection of the hosts (48). Several regulatory mechanisms have been described for TLRs including soluble and decoy factors, membrane-associated protein regulators, negative regulators of the adaptor complex, and microRNA (3). To assess the expression of these negative regulators of TLRs, we first cloned cDNAs corresponding to these proteins in the bovine (19). We demonstrated the expression of A20-binding inhibitor of nuclear factor kappa B activation 3 (ABIN-3); B-cell lymphoma 3-encoded protein (Bcl-3); interleukin-1 receptor-associated kinase M (IRAK-M); single immunoglobulin IL-1-related receptor (SIGIRR); toll interacting protein (Tollip); and mitogen-activated protein kinase 1 (MKP-1) in BIE cells (19). Consequently, the effect of *L. casei* OLL2768 on the expression of these negative regulators of the TLRs signaling was next evaluated. *L. casei* OLL2768 is able to up-regulate Tollip and Bcl-3 in BIE cells, and in this way to negatively regulate TLR4 signaling (19). Tollip is able to suppress the activity of IL-1 receptor-associated kinase (IRAK), and inhibit TLR4-triggered NF-κB and MAPK signaling pathways (49, 50). It was showed that stimulation of IECs with a TLR ligand, such as LPS, induces a state of hyporesponsiveness through up-regulation of Tollip that limits pro-inflammatory responses triggered by a second challenge with the same or another TLR ligand (50). The expression of Tollip was reported in bovine mammary epithelial cells (51). The work showed that expression of Tollip was increased in response to LPS, suggesting that the bovine mammary epithelium possesses the necessary immune repertoires required to regulate TLR4 activation. Recently, it was demonstrated by Fu et al. (52) that Tollip is significantly up-regulated in bovine endometrial epithelial cells after the stimulation with LPS, and that this up-regulation of Tollip was necessary for the regulation of the overexpression of NF-κB and the protection against the inflammatory damage. On the other hand, the Bcl-3 is a nuclear protein and member of the NF-κB family. Bcl-3 is able to stabilize repressive NF-κB homodimers in a DNA-bound state, and in this way prevents the binding of transcriptionally active dimers. Therefore, Bcl-3 functions as an inhibitor of NF-κB activity. In recent years, a role of Bcl-3 has been revealed in LPS tolerance via its ability to stabilize the p50 homodimer, and thus, has been identified as a negative regulator of TLR4 signaling (53). Furthermore, by selectively affecting chromatin remodeling, Bcl-3 mediates repression of pro-inflammatory genes, and also facilitates the expression of the anti-inflammatory gene *IL-10* (54). Therefore, the induction of Bcl-3 and Tollip by *L. casei* OLL2768 in BIE cells is important in establishing tolerance against heat-stable ETEC MAMPs.

**Figure 2:**
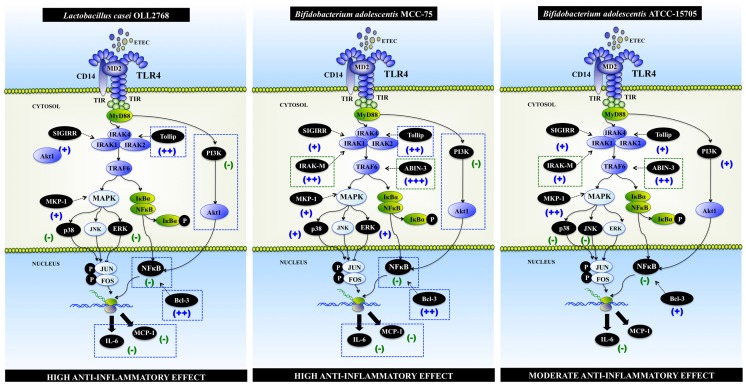
**Modulation of toll-like receptor 4 (TLR4) signaling pathway by *Lactobacillus casei* OLL2768, *Bifidobacterium adolescentis* MCC-75, and *Bifidobacterium adolescentis* ATCC15705 in bovine intestinal epithelial epitheliocytes (BIE cells)**. Anti-inflammatory immunobiotic strains up-regulate the expression of TLR negative regulators, reduce the activation phosphatidylinositol 3-kinase (PI3K), mitogen-activated protein kinase (MAPK), and nuclear factor κB (NF-κB) pathways; and diminish the production of inflammatory mediators by BIE cells.

It is not possible to give a precise molecular mechanism for the anti-inflammatory action of *L. casei* OLL2768 on BIE cells at present. However, it could be hypothesized that interaction of *L. casei* OLL2768 with BIE cells through one or more PRRs induces the up-regulation of the negative regulators Bcl-3 and Tollip, which reduce the production of inflammatory mediators in response to heat-stable ETEC MAMPs (Figure 2). One of the possible PRR involved in *L. casei* OLL2768 immunoregulatory capacities could be TLR2. Studies with the TLR2 ligand Pam3CSK4 in BIE cells demonstrated that the treatment with the TLR2 agonist up-regulate the expression of Tollip and reduce activation of NF-κB and p38 MAPK pathways (19). However, further research is needed to resolve which PRR is activated by *L. casei* OLL2768 for the induction of negative regulators.

## Regulation of Inflammation in BIE Cells by Immunobiotic Bifidobacteria

Members of the genus *Bifidobacterium* are considered to be important constituents of the microbiota of animals, from insects to mammals. They are gut commensals extensively used by the food industry as probiotic microorganisms, since some strains have been shown to have specific beneficial effects. Bifidobacteria are able to prevent or alleviate infectious diarrhea through their effects on the immune system and resistance to colonization by pathogens. In addition, some bifidobacteria strains have potent anti-inflammatory capacities that could be used to reduce inflammatory-intestinal damage. *Bifidobacterium animalis* strain AHC7 decrease NF-κB activation in mice infected with *S. typhimurium*. *B. animalis* AHC7 consumption in this mouse model was associated with protection against inflammatory damage through modulation of secreted IL-10 and IL-12p70 and enhancement of Foxp3 expression in naïve T cells (55). In line with these results, it was showed that *Bifidobacterium bifidum* W23 was able to induce a suppression of IL-8 synthesis by Caco-2 cells challenged with *S. enterica* serovar enteritidis, and that the protective role of this probiotic strain was mediated, at least in part, via Hsp70 expression (56). Moreover, it was recently reported that *Bifidobacterium adolescentis* FRP 61, *Bifidobacterium longum* FRP 68 and FRP 69, and *Bifidobacterium breve* FRP 334 significantly reduced IL-8 production by HT-29 cells challenged with *S. typhimurium* (57). Others studies evaluating the effect of bifidobacteria in intestinal Caco-2 cells showed that *B. animalis* MB5 avoid cytokine deregulation upon ETEC challenge by inducing upregulation of IL-1β and TNF-α, and the down-regulation of TGF-β expression (58, 59). Additionally, we demonstrated in porcine IECs cells that treatment with *B. breve* MCC-117 significantly reduced the expression of inflammatory cytokines in response to heat-stable ETEC MAMPs. Moreover, studies with porcine immune cells showed that *B. breve* MCC-117 was able to reduce the levels of IFN-γ in CD4^+^ and CD8^+^ lymphocytes and improved IL-10 levels in CD4^+^CD25^high^Foxp3^+^ lymphocytes (14). These are among several other studies that clearly showed that bifidobacteria are highly effective in regulating pathogenic inflammation in the gut. Therefore, we next aimed to select potential immunomodulatory bifidobacteria able to beneficially modulate the inflammatory response in BIE cells.

The potential use of bifidobacteria as probiotic for cattle is supported by some new reports indicating the presence of these bacteria in young calf intestines and the fact that their presence in high numbers is associated with good health status of the host (60). Therefore, some bifidobacteria strains, previously selected in our porcine systems, were used to evaluate their anti-inflammatory capacities in heat-stable ETEC MAMPs-challenged-BIE cells (61). Similarly to the effect of *L. casei* OLL2768, some bifidobacteria were able to reduce the production of inflammatory mediators triggered by TLR4 activation in BIE cells. Considering their ability to reduce the expression of IL-6 and MCP-1, bifidobacteria strains were divided in the following two groups: (1) strains able to reduce both IL-6 and MCP-1 (*B. adolescentis* MCC-75 and *B. breve* MCC-117) and (2) strains able to reduce only IL-6 (*B. longum* BB536, *B. adolescentis* ATCC15705 and *Bifidobacterium infantis* MCC-1021) (61).

As described for the immunoregulatory *L. casei* OLL2768 strain, we also aimed to evaluate signaling pathways and TLR negative regulators expression in BIE cells after the treatment with bifidobacteria belonging to the two functional groups defined by our studies. Then, we selected *B. adolescentis* MCC-75, *B. breve* MCC-117 (strains with high anti-inflammatory capacities), and *B. adolescentis* ATCC15705 (strain with moderate anti-inflammatory capacity) for further experiments. Activation of MAPK, NF-κB, and phosphatidylinositol 3-kinase (PI3K) pathways, and changes in the expression of TLR negative regulators in MCC-75-, MCC-117- and ATCC15705-treated BIE cells were then studied. We found that each bifidobacteria strain induces unique changes in TLR4 signaling in bovine IECs (61) (Figure 2).

As mentioned before, several negative regulatory mechanisms control TLRs-mediated inflammatory responses and restore immune system balance in the gut. Although the NF-κB-dependent gene expression is critical to the induction of an efficient immune response, excessive, or prolonged NF-κB signaling can contribute to the development of several inflammatory diseases. Therefore, this signaling transduction pathway has to be tightly regulated by several intracellular proteins. The ubiquitin-editing enzyme A20 is key regulator of the TLRs signaling. It was showed that A20 deficiency in IECs renders mice sensitive to TNF-α-induced lethal inflammation (62, 63). Moreover, it was reported that A20 is an early response negative regulator of TLR4 and TLR5 signaling in IECs that functions during intestinal inflammation to control the innate immune system (64). In addition, the A20-binding inhibitor of NF-κB activation (ABIN) is LPS-inducible proteins that negatively regulate NF-κB activation in response to TNF-α and LPS (65). ABINs have been described as three different proteins (ABIN-1, -2, and -3) that bind A20. Overexpression of ABINs inhibits NF-κB activation by TNF-α and several other stimuli. Similar to A20, ABIN-3 expression is NF-κB dependent, implicating a potential role for the A20/ABIN complex in the negative feedback regulation of NF-κB activation (66). Therefore, the induction of A20/ABIN complex by bifidobacteria in BIE cells is important in establishing tolerance against heat-stable ETEC MAMPs. This is in line with our previous reports in porcine IECs. In our works in the porcine systems, we showed that the bifidobacteria strains with the highest capacity to downregulate the expression of inflammatory cytokines in response to heat-stable ETEC PAMPs were also able to up-regulate A20. In fact, the most potent anti-inflammatory bacteria evaluated in our laboratory, bifidobacteria strains BB536 and M-16V and *L. jensenii* TL2937, strongly up-regulated the ubiquitin-editing enzyme A20 [for a review see Ref. (3)].

Bcl-3 protein functions as an inhibitor of NF-κB activity as mentioned before. In addition, SIGIRR, Tollip, and IRAK-M are also known to be expressed at high levels in IECs, and to thereby contribute to the hyporesponsiveness of IECs to commensals (64, 67, 68). Therefore, induction of these five negative regulators by bifidobacteria in BIE cells may be important for establishing tolerance against heat-stable ETEC MAMPs (Figure 2). Moreover, the fold expression increase of the negative regulators of the TLRs signaling should be also important since the levels of ABIN-3, IRAK-M, and Bcl-3 were significantly higher in *B. breve* MCC-117- and *B. adolescentis* MCC-75-treated BIE cells when compared with BIE cells treated with the moderate anti-inflammatory strain *B. adolescentis* ATCC15705 (61).

On the other hand, the MAPK pathway is involved in the upregulation of several inflammatory genes, and MKP-1 plays a role in the inhibition of pro-inflammatory mRNA expression, because it can inactivate MAPK pathway (69). Therefore, we expected that bifidobacteria with high anti-inflammatory activity significantly up-regulate MKP-1 expression and reduce MAPK activation as we have observed with other anti-inflammatory immunobiotic strains (3). However, when ERK, p38, and JNK MAPK activation and MKP-1 expression were studied in BIE cells treated with bifidobacteria, we found that *B. adolescentis* MCC-75 and *B. breve* MCC-117 activated ERK MAPK pathway and only moderately up-regulated MKP-1. On the contrary, *B. adolescentis* ATCC15705 strongly increased expression of MKP-1 and inhibit p38 and JNK pathways (61). It is known that the ERK pathway play key regulatory functions in a diverse spectrum of biological processes such as cell proliferation, differentiation, survival, and motility (70). It was also reported that TGF-β induces ERK activity in IECs and this TGF-β/ERK interaction regulates genes that are crucial for cell growth, migration, and survival of IECs (71, 72). In fact, treatment with TGF-β prevents mucosal-injury, enhances p-ERK and β-catenin, induces enterocyte proliferation, inhibits enterocyte apoptosis, and improves intestinal recovery following methotrexate-induced intestinal-mucositis in rats (73). Moreover, TGF-β increases protein levels, collagen I, TGF-β of type-1 inhibitor of plasminogen activator, and the TGF-β-converting enzyme furin in various IEC lines via ERK (74) indicating an important immunoregulatory role of the ERK pathway in maintaining homeostasis in IECs. Therefore, the activation of the ERK pathway by *B. adolescentis* MCC-75 and *B. breve* MCC-117 during ETEC MAMPs-mediated inflammation could have an important protective role against inflammatory damage (61).

Our studies also demonstrated that both *B. adolescentis* MCC-75 and *B. breve* MCC-117 were able to inhibit PI3K pathway in heat-stable ETEC MAMPs-challenged-BIE cells. It is known that PI3K regulates TLR signaling in both positive and negative ways. By mutating specific tyrosine residues in the cytosolic domain of TLR2, it was showed that there is a loss in the capacity of p85 to associate with this receptor and in the ability of TLR2 to activate NF-κB pathway. Furthermore, inhibition of PI3K during TLR2 stimulation has been shown to reduce NF-κB activation (75). On the contrary, studies in PI3K or p85a deficient mice showed that PI3K negatively regulates TLR signaling (76, 77). Then, it is well established that PI3K could affect TLRs signaling pathways in different ways and that it effect depends on cell type and readout. In our studies, we showed that stimulation of BIE cells with heat-stable ETEC MAMPs activated PI3K pathway, indicating that PI3K is positively involved in TLR4 signaling in BIE cells (Figure 1B). Moreover, we demonstrated that bifidobacteria able to reduce activation PI3K pathway were the strains with the highest anti-inflammatory activity (61) (Figure 2).

As mentioned before, our works evaluating the immunoregulatory activity of immunobiotics demonstrated that the upregulation of some regulatory cytokines and down-regulation of inflammatory mediators is dependent of TLR2 activation (15, 16). Therefore, we also investigated the role of TLR2 in the immunoregulatory effects of *B. adolescentis* MCC-75, *B. breve* MCC-117, and *B. adolescentis* ATCC15705 by using anti-TLR2 blocking antibodies (61). It was showed that the reduction of IL-6 induced by bifidobacteria in ETEC MAMPs-challenged-BIE cells was abolished when anti-TLR2 antibodies were used. This is in line with other reports conferring a key role to TLR2 in the recognition of bifidobacteria, which possess anti-inflammatory activities (78–80). It is known that stimulation of TLR2 is able to induce tolerance against a subsequent LPS challenge (81). Therefore, it is possible that bifidobacteria could induce this type of cross-tolerance in BIE cells through their interaction with TLR2. In addition, we showed that the reduction of MCP-1 levels after challenge of BIE cells was not abolished when anti-TLR2 antibodies were used. This finding indicates that additional PRRs may be involved in the anti-inflammatory effects of *B. adolescentis* MCC-75, and *B. breve* MCC-117 in BIE cells.

## Conclusion

The knowledge of the cellular and molecular interactions of human IECs with commensal and probiotic bacteria is rapidly progressing. An exciting possibility is that similar systems developed for the bovine host could serve as a platform for medicine and research. However, to achieve this goal, bovine IECs cultures must be enhanced and improved to allow that functional assays can be performed. Our research work has demonstrated that the BIE cell line is a useful *in vitro* tool for the study of TLR4-induced inflammatory responses in the bovine intestinal epithelium. We have also demonstrated that BIE cells could be used for a rapid screening and selection of potential immunobiotic bacteria as well as for studying the molecular mechanisms involved in their beneficial protective activity.

Despite the unique effect of each lactobacilli or bifidobacteria strain, some general conclusions can be made when comparing the effect of the two different immunoregulatory groups: high anti-inflammatory activity (*L. casei* OLL2768, *B. adolescentis* MCC-75, and *B. breve* MCC-117) and moderate anti-inflammatory activity (*B. adolescentis* ATCC15705) (Figure 2): (1) anti-inflammatory capacity in BIE cells is strain dependent, as demonstrated by the differential effect induced by each strain, even those of the same specie (*B. adolescentis* MCC-75 and *B. adolescentis* ATCC15705). (2) The upregulation of TLR negative regulators and the intensity of that upregulation would be related to the different immunomodulatory capacity of each immunobiotic strain. Notably, upregulation of Tollip and Bcl-3 seems to be related to a high anti-inflammatory capacity. (3) The inhibition of PI3K pathway would be related to the high anti-inflammatory effect of immunobiotics in BIE cells. (4) The balance between MAPK activation and MKP-1 upregulation would be related to the anti-inflammatory effect of bifidobacteria in BIE cells. (5) The anti-inflammatory effect of immunobiotics in BIE cells is partially dependent on TLR2. Further research is needed to resolve which other PRR is involved in the immunoregulatory effects. In addition, one general conclusion can be made when comparing the effect of the two different immunoregulatory groups of bifidobacteria (Figure 2). (6) The upregulation of IRAK-M and ABIN-3 and the intensity of that upregulation would be related to the different immunomodulatory capacity of each bifidobacteria strain.

We believe that studies in BIE cell would provide useful information that may help in the near future to develop new functional feeds able to beneficially modulate the mucosal immune system in the bovine host. In line with this, we showed in our studies that the immunoregulatory strains *L. casei* OLL2768, *B. adolescentis* MCC-75, and *B. breve* MCC-117 are able to functionally modulate BIE cells by attenuating TLR4-induced NF-κB, MAPK, and IP3K activation and inflammatory cytokines production. Then, OLL2768, MCC-75, and MCC-117 strains would be good candidates for *in vivo* evaluation of the protective effect of immunobiotics against inflammatory damage induced by bovine intestinal pathogens or their associated PAMPs. We also believe that the immunobiotic application in cattle could contribute to produce safety animal foods via improving bovine health.

## Conflict of Interest Statement

The authors declare that the research was conducted in the absence of any commercial or financial relationships that could be construed as a potential conflict of interest.
